# Investigation of the Co-Movement Relationship between Medical Expenditure and GDP in Taiwan-Based on Wavelet Analysis

**DOI:** 10.3390/ijerph16245095

**Published:** 2019-12-13

**Authors:** Hsin-Pei Hsueh, Chien-Ming Wang, Cheng-Feng Wu, Fangjhy Li

**Affiliations:** 1 School of Finance, Hubei University of Economics, Wuhan 430205, China; xuexinbei@hbue.edu.cn; 2 School of Economics and Trade, Hubei University of Economics, Wuhan 430205, China; wangjianming@hbue.edu.cn; 3 Research Center of Hubei Logistics Development, Hubei University of Economics, Wuhan 430205, China; wuchengfeng@hbue.edu.cn; 4 School of Business Administration, Hubei University of Economics, Wuhan 430205, China

**Keywords:** gross domestic product, medical expenditures, Wavelet analysis, co-movement relationship, health insurance

## Abstract

In this study, using the medical expenditures of the Taiwanese government and gross domestic product (GDP) as variables, the wavelet analysis method was used to empirically study the correlations and lead-lag relationships in quarterly data in the period from 1996 to 2016. In addition, the dependent population of the insured was used as the control variable. Results: After the dependent population was included as a control variable, there was a period of low- frequency (one to four years, short-term) linkage correlation, as well as a period of high- frequency (four to eight years, long-term) linkage correlation. In addition, for more than eight years, there was a high degree of linkage correlation, indicating that the linkage between medical expenditures and GDP occurred over the long- term. Moreover, since medical expenditures positively affected GDP, one-way causality was observed. However, after 2008, regardless of whether long or short- term was examined, there was almost no linkage correlation. Before 2008, the medical expenditures of the government were positively correlated with economic growth. After 2008, this effect had already disappeared. The universal health insurance system has long been denounced as a waste of medical resources. The government needs to find a new solution.

## 1. Introduction

In Taiwan’s universal health insurance system, the insured include all nationals and foreigners who have residence permits. The policy was formed with good intentions to help vulnerable groups. It also allows people with major illnesses and chronic diseases to no longer worry about huge medical expenses. The Kuomintang government enacted the universal health insurance system was enacted by the Kuomintang Government after a vote. At one time, more than 8 million retired elderly people, children, housewives, unemployed, and other uninsured people were able to receive health insurance and healthcare security. Internationally, the medical health insurance system in Taiwan has also received wide acclaim, and the domestic populace’s degree of satisfaction with it was higher than 70%. Social incidents in the past, such as acute or critical illnesses that necessitated patients raising money, or in which long-term medical expenses of patients with chronic illnesses harmed the families, all came to an end.

The universal health insurance system has long been denounced as a waste of medical resources, and the waste must be immediately stopped. The health insurance system was formed with good intentions to help vulnerable groups and patients who have acute, critical, and chronic illnesses, so that they do not need to worry about treating their illnesses. Insurance was originally designed to transfer risk and prevent losses that are caused by accidents or diseases.

However, since universal health insurance was implemented in 1995, the finances of the program have shifted from bad to worse. This is because the number of unnecessary and repeated visits to hospitals and clinics has increased. It is critical to emphasize that medical attention should be sought only when there is a need.

The convenience of medical treatment in Taiwan resulted in increasingly high government medical expenditures, wasted medical resources, and problems related to health insurance restructuring. These issues were all denounced by the people, and the possibility of bankrupting the system was raised. Therefore, the Taiwanese government urgently needs to consider improvements to this system.

## 2. Literature Review

In foreign literature, early studies on the effect of government medical expenditures on gross domestic product (GDP) mainly concentrated on the relationship between medical expenditures and GDP. Moreover, most of the studies supported positive correlation correlation between government medical expenditures and GDP growth. Abel-Smith [1,2] was the first to discover that the ratio of medical expenditure to GDP was positively correlated with GDP. Thus, higher GDP resulted in a higher ratio of medical expenditures to GDP, indicating that GDP was an important factor that determined government medical expenditures. Later, the study by Kleiman [3] showed that government revenues also had a significant effect on government medical expenditures and that per capita GDP [4] was a major influencing factor of government medical expenditures. Many subsequent empirical studies also came to this conclusion. For example, Gbesemete and Gerdtham [5] found that, in some African countries, per capita GDP was the biggest factor influencing per capita medical expenditures. The analysis by Hitris and Posnett [6], on the Organization for Economic Cooperation and Development (OECD) countries, also found an extremely strong correlation between per capita GDP and medical expenditures. Gyimah-Brempong [7] drew the same conclusion in a subsequent study.

When studying the effects of government medical expenditures on economic development, most scholars believed that government medical expenditures had a positive effect on GDP growth. Gerdtham [8] found that in some OECD countries, per capita income had a significant and positive effect on government medical expenditures. Azeem [9] studied Pakistan and found that increasing medical expenditures could increase economic development. Gerdtham and Löthgren [10] used the unit root test to conduct a cointegration analysis of 21 OECD countries to find the long-term relationship between medical expenditures and GDP.

In studies that targeted the relationship between GDP and government medical expenditures, some scholars found that government medical expenditures had neither a significant influence on, nor a negative correlation with, GDP growth. Hansen and King [11] analyzed 20 OECD countries from 1960 to 1987 and found that there was no long-term relationship between medical health insurance expenditures and GDP in OECD countries, thereby not supporting the effect of GDP on medical health insurance expenditures. Similarly, Devlin and Hansen [12] analyzed 20 OECD countries from 1960 to 1987 to test the Granger causality between medical health insurance expenditures and GDP. They found that six countries had no evidence to support that there was causality between the two factors. In eight countries, medical health insurance expenditures affected GDP; in another eight countries, GDP affected medical health insurance expenditures; and in two countries, the two affected each other.

There were also a few scholars who supported that the two were negatively correlated. For example, Mohapatra et al. [13] used data from 16 main Indian states to divide government medical expenditures into profitable and capital-related medical expenditures. The results revealed that there was no influence on the national economy, but there was an effect on the states—capital government medical expenditures were found to affect the states’ long-term GDP development.

## 3. Research Method and Data

In this section, we introduce the wavelet analysis method used in this study to verify the linkage between the medical expenditures of the Taiwanese government and gross domestic product (GDP).

### 3.1. Research Method

Wavelet analysis is a mathematical tool that has emerged in the last decade. Wavelet analysis, is also known as wavelet transform. Haar in 1910 proposed the concept of. French physicist J. Morlet then improved the traditional Fourier transform in 1984 to analyze the local properties of seismic waves. This wavelet signal was used to introduce signal analysis and decompose signals. The wavelet transform is the result of wavelet analysis. Then, physicist A. Grossman began to study Morlet’s scaling and the $\left\{a-\frac{1}{2}\Psi \left(\frac{x-b}{a}\right):a,\;b\in R\right\}$ translation system for a certain function, which was the beginning of wavelet analysis. It became an important discovery and breakthrough after the 1822 “Fourier transformation” analysis, which was quickly applied to many fields.

However, the “Fourier transformation” cannot identify the structural mutation of the time series at the local frequency, and thus lacks good time domain localization properties; In addition, the Fourier transform requires the time series to have good stability. The economic and financial time series are mostly unsteady.

The wavelet transform can overcome the shortcomings of the Fourier transform, and it has also evolved. Because of the time domain and frequency-domain localization properties, it can be superior to the “Fourier transform” when dealing with non-stationary time series, as well as time domain and frequency-domain localization information. It can be used to analyze irregular waveforms and to better deal with time series information for abrupt structural changes in non-stationary time series.

The following are four wavelet analysis tools: continuous wavelet transform, wavelet power spectrum, wavelet correlation, and phase difference.

#### 3.1.1. Continuous Wavelet Transform

Continuous wavelet transform can construct time-frequency signals with good time domain and frequency-domain localization. The information that is difficult to see in the time series is revealed in the time domain and the frequency-domain. The wavelet transform has (1) discrete wavelet transform and (2) continuous wavelet transform. The discrete wavelet transform is a special case, which is not introduced. The introduction is mostly a continuous wavelet transform used on financial data. 

It can be listed as follows:

$$W_{x}\left(\tau ,s\right)=\int_{-\infty }^{+\infty }\chi \left(t\right)\psi_{\tau ,s}^{*}\left(t\right)dt$$

where * represents a complex conjugate, i.e., $\psi_{\tau ,s}^{*}\left(t\right)$ is a complex conjugate function of the $\psi_{\tau ,s}$ function, and $\psi_{\tau ,s}$ is the base wavelet function.

As mentioned above, it is a sequence of functions obtained by the mother wavelet function after the warping translation. ψ(t) represents the mother wavelet function. The base wavelet function ψ(t) and the mother wavelet function ψ(t) can be listed as:

$$\psi_{\tau ,s}=\frac{1}{\sqrt{s}}\psi \left(\frac{t-\tau }{s}\right)$$

where *s* is a parameter table mother wavelet expansion; *τ* is a parameter table mother wavelet translation; and the $\frac{1}{\sqrt{s}}$ factor corresponds to the frequency. Different values of *s* will produce different mother wavelet functions: s>1 is the narrow mother wavelet; s<1 is the wide mother wavelet. The narrow mother wavelet is for the high-frequency part with a short duration in the sequence, and the wide mother wavelet has a good expression for the low-frequency part with a longer duration in the sequence. Since the s and τ values change continuously, ψ(t) is called the continuous mother wavelet function, and $W_{x}\left(\tau ,s\right)$ is called the continuous wavelet transform function.

#### 3.1.2. Wavelet Power Spectrum

The wavelet power spectrum of a single time series χ(t) becomes the wavelet self-power spectrum, which is used to measure the volatility of χ(t) in the time domain and frequency domain combination, as follows:

$$\sigma_{\chi }^{2}=\int_{0}^{+\infty }\int_{-\infty }^{+\infty }{\left|W_{\chi }\left(\tau ,s\right)\right|}^{2}\frac{d_{\tau }d_{s}}{s^{2}}.$$

The cross-wavelet power spectrum of the two time series χ(t) and y(t) has been defined as the wavelet self-power spectral product of χ(t) and y(t), namely: ${\left|W_{x,y}\left(\tau ,s\right)\right|}^{2}={\left|W_{x}\left(\tau ,s\right)\right|}^{2}{\left|W_{y}^{*}\left(\tau ,s\right)\right|}^{2}$. The cross-wavelet power spectrum can be used to measure the local co-shift between χ(t) and y(t) in the combination of time domain and frequency domain.

#### 3.1.3. Wavelet Coherency Coefficient

Based on the wavelet power spectrum, the ratio between the cross-wave power spectrum of the time series χ(t) and y(t) and the respective wavelet power spectra can be further used to measure the ratio between χ(t) and y(t). The local correlation in the time domain and the frequency domain is called the wavelet correlation coefficient, which is represented by $R_{n}^{2}\left(s\right)$:

$$R_{n}^{2}\left(s\right)=\frac{{\left|S(s^{-1}W_{x,y}\left(\tau ,s\right))\right|}^{2}}{S(s^{-1}{\left|W_{x}\left(\tau ,s\right)\right|}^{2})S(s^{-1}{\left|W_{y}\left(\tau ,s\right)\right|}^{2})}.$$

In the above formula, S is a smoothing factor for performing time-frequency normalization processing. The value of $R_{n}^{2}\left(s\right)$ is between 0 and 1. When $R_{n}^{2}\left(s\right)$ is equal to 1, it means that χ(t) and y(t) are completely correlated; when $R_{n}^{2}\left(s\right)$ is equal to 0, it means that there is no connection between χ(t) and y(t).

#### 3.1.4. Phase Difference

**Figure 1:**
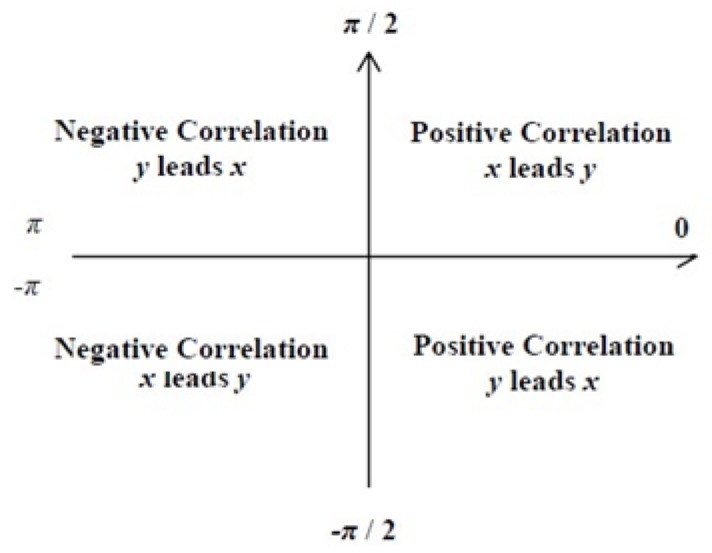
Phase Difference Icon.

Figure 1 shows the leading edge (causal) relationship of the phase difference diagram:

In [14], it is proved that the phase difference between the time series χ(t) and y(t) can be used to measure the lead-lag relationship between the two at a specific time frequency. The phase difference is defined as the ratio of the imaginary part ℑ to the real part ℜ of the cross wavelet power $W_{x,y}\left(\tau ,s\right)$, expressed as follows:

$$\phi \left(\tau ,s\right)=\tan^{-1}\left(\frac{ℑ\left\{\left(W_{xy}\left(\tau ,s\right)\right)\right\}}{ℜ\left\{\left(W_{xy}\left(\tau ,s\right)\right)\right\}}\right)$$

ϕ(τ,s)∈[−π, π] represents the correlation between χ(t) and y(t) at a particular frequency:

(1) When $\phi \left(\tau ,s\right)\in \left(0,\frac{\pi }{2}\right)$, then χ(t) and y(t) are positively correlated, and χ(t) leads y(t).
(2) When $\phi \left(\tau ,s\right)\in \left(\frac{\pi }{2},\pi \right)$, then χ(t) and y(t) are negatively correlated, and y(t) leads χ(t).
(3) When $\phi \left(\tau ,s\right)\in \left(0,-\frac{\pi }{2}\right)$, then χ(t) and y(t) are positively correlated, and y(t) leads χ(t).
(4) When $\phi \left(\tau ,s\right)\in \left(-\frac{\pi }{2},-\pi \right)$, χ(t) and y(t) are negatively correlated, and χ(t) leads y(t).
(5) When ϕ(τ,s)=0, then χ(t) and y(t) exhibit a complete positive correlation, i.e., have covariance.
(6) When ϕ(τ,s)=π, then χ(t) and y(t) exhibit a complete negative correlation.

### 3.2. Data

Taiwan was used as the sample country in this study. Health insurance medical expense expenditures and GDP were used as the variables, and the dependent population of the insured was used as the control variable. The dependent population consisted of spouses and immediate relatives who were not employed, most of whom were underage children and retired elderly people 65 years of age or above. This population has no income and accounts for a high proportion of medical expenditures. The data period was from the first quarter of 1996 to the fourth quarter of 2016, which resulted in a total of 84 quarters of data. The GDP data were sourced from the Directorate General of Budget, Accounting, and Statistics of Taiwan, which is a branch of the Executive Yuan, The data on health insurance, medical expense expenditures, and the dependent population of the insured were sourced from the Bureau of National Health Insurance of Taiwan.

## 4. Empirical Results

### 4.1. Narrative Statistics

The narrative statistics is shown in Table 1.

### 4.2. Wavelet Analysis

The following is the wavelet analysis between health insurance medical expenditures in Taiwan and GDP.

Results: Before the control variable was included, the whole sample period had only high frequency (one-year, short-term) linkage correlation, and the high- frequency (four- to eight-year, long-term) effect was very weak.

After the control variable was included, there was low- frequency (one- to four-year, short-term) linkage correlation, and there was two-way causality. The high- frequency effect (four to eight years or more than eight years) had a high degree of linkage correlation, and there was one-way causality in which medical expenditures positively affected GDP. However, after 2008, regardless of whether the correlation was long or short- term, there was almost no linkage correlation. Therefore, the Taiwanese government should consider the appropriateness of medical expenditures.

**Figure 2:**
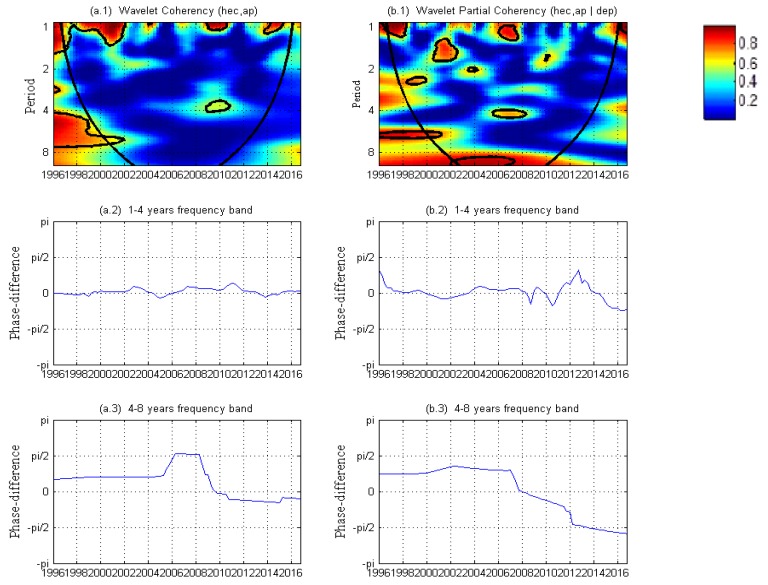
Linkage analysis between health insurance medical expenditures in Taiwan and GDP with the control variable of the dependent population. (**a.1**) Wavelet Coherency, (**a.2**) 1–4 year frequency band, (**a.3**) 4–8 year frequency band, (**b.1**) Wavelet Partial Coherency, (**b.2**) 1–4 year frequency band, (**b.3**) 4–8 year frequency band.

As shown in Figure 2(a.1), in terms of the correlation between Taiwan’s health insurance medical expenditures and GDP, short-term (one- to four year) effects were found from 1997 to the end of 1999, at the end of 2001, and from 2009 to the middle of 2010. In 2014, there was a significant positive correlation (correlation coefficient > 0.7), and the significant correlation was within one year (an extremely short-term frequency). There was a long-term (four- to eight-year) positive correlation only in 2001 (correlation coefficient > 0.4), and there was no linkage correlation for the rest of the period.

After adding the control variable, as in Figure 2(b.1), short-term (one- to four year) effects were found from 1997 to the end of 1999, in the middle of 2000 and 2002, and from 2006 to the third quarter of 2007, and there was a more significant positive correlation (correlation coefficient > 0.6). Positive long-term (four to eight years and more than eight years) correlations were found in 2000 and from 2002 to the beginning of 2008 (correlation coefficients > 0.7 and 0.5, respectively). After 2008, there was almost no linkage correlation in the long or short- term.

In Figure 2(a.2), short-term (one- to four year) effects were found from 1997 to the end of 1999, and at the end of 2001, medical expenditures were positively correlated and covaried with GDP. From 2009 to the middle of 2010, medical expenditures and GDP were positively correlated, and medical expenditures led GDP. In 2014, medical expenditures were positively correlated and covaried with GDP, and the period of the significant correlation was within one year (an extremely short-term frequency).

For Figure 2(a.3), the long-term (four to eight years) phase difference showed that there was a positive correlation (correlation coefficient >0.4) only in 2001, and medical expenditures led GDP.

After including the control variable of the dependent population, in Figure 2(b.2), in the short term (one to four years) from 1997 to the end of 1999, medical expenditures were positively correlated and covaried with GDP. From the middle of 2000 to the middle of 2002, medical expenditures and GDP were positively correlated, and medical expenditures led GDP (medical expenditures positively affected GDP). From 2006 to the third quarter of 2007, medical expenditures and GDP were positively correlated with medical expenditures leading GDP. Therefore, the short-term (one to four years) phase difference showed that the two had mutual causality. After 2008, there was almost no linkage correlation.

In Figure 2(b.3), positive long-term (four to eight years and more than eight years) correlations were found in 2000 and from 2002 to the beginning of 2008, and medical expenditures led GDP. After 2008, there was almost no linkage correlation, regardless of long or short-term.

To summarize, for the whole sample period, the correlation between medical expenditures and GDP was positive.

The short-term (one to four years) phase difference showed that, before including the control variable, medical expenditures and GDP were positively correlated, and there was covariation or a positive correlation with medical expenditures leading GDP. After including the control variable, medical expenditures and GDP were positively correlated and had mutual causality.

The long-term phase difference (four to eight years and more than eight years) showed that regardless of whether the control variable was included, medical expenditures and GDP were positively correlated with medical expenditures leading GDP. However, after the control variable was added, there was almost no linkage correlation after 2008, regardless of the time period (long or short-term).

## 5. Conclusions

The economic growth rate of Taiwan has recently decelerated. In particular, since 1980, the mean real GDP growth rate has decreased, and the growth rate of medical expenditures has peaked. The empirical results showed that before 2008, increasing medical expenditures for the Taiwan government would increase Taiwan’s GDP. Medical expenditures are government expenditures, but those applying for medical expenses were major public and private hospitals, regional hospitals, private clinics, etc. Medical expenditures ultimately increased GDP, which may have been influenced by differences between the inflated reported figures and differences in drug prices. Furthermore, the profit gaps between various medical units were very large.; therefore, high medical expenditures can result in economic benefits.

Before 2008, Taiwan did not have a relative increase in medical expenditures when only GDP increased (the economic environment improved); instead, medical expenditures increased Taiwan’s GDP. Moreover, the GDP growth that was caused by high medical expenditures was truly caused by wasted domestic fund transfers, in which the government and its citizens supported the profits of the medical industry by increasing the industry’s surpluses or undeserved profits (this was due to wasted medical resources.). However, after 2008, this effect had already disappeared. The health insurance system restructuring in 2008 may be one of the reasons.

The universal health insurance system has long been denounced as a waste of medical resources, and the waste must be immediately stopped. The health insurance system was formed with good intentions to help vulnerable groups and patients who have acute, critical, and chronic illnesses, so that they do not need to worry about treating their illnesses. All people should be willing to help others. Insurance was originally designed to transfer risk and prevent losses that are caused by accidents or diseases. Because there are no longer paid health insurance premiums, the number of unnecessary and repeated visits to hospitals and clinics has increased. It is critical to emphasize that medical attention should be sought only when there is a need.

The empirical results showed that prior to 2008, the medical expenditures of the government were positively correlated with economic growth; i.e., they enhanced economic growth. If reduced medical expenditures were recommended at this time to balance the profits and losses in the health insurance system, GDP growth would have also decreased synchronously. However, after 2008, this effect disappeared, and there was no longer any linkage correlation between medical expenditures and GDP. The continued expansion of medical expenditures no longer increased GDP in Taiwan. Therefore, the government should adopt corresponding measures to prevent wasted medical resources and explore the effectiveness of the health insurance system after the health insurance restructuring in 2008, as well as the installation of the present health insurance system. This work provides corresponding thoughts and recommendations for the Taiwanese government.

The Taiwanese government should reconsider the appropriateness of the current health insurance system; revise the items that are covered by health insurance, such as copayments for doctor’s visits and the health checkup cycle; eliminate repetition and waste; and update other response measures, that have become pressing policy issues.

Policy budget: Before 2008, the increase in government medical expenditure also brought about the growth of GDP, which was positively related in the same direction. However, after 2008, this effect disappeared. It means that medical expenditure should not be allowed to grow again, because it has no positive effect on GDP, it should have been tightened. The government can increase the amount of out-of-pocket expenses for medical treatment, or the premiums paid by the public, to solve the medical waste caused by medical convenience in Taiwan.

## Figures and Tables

**Table 1:** Narrative statistics.

Variable	HEC	GDP	DEP
Mean	107,728.8	3,123,724	8,652,338
Median	106,761.5	3,113,779	8,733,195
Max	171,831	4,443,027	9,221,601
Min	52,331	1,929,854	8,054,069
Std. Dev	33,051.01	658,906.2	390,786.1
Skewness	0.141683	0.129568	−0.217136
Kurtosis	1.889501	2.033702	1.567612
Jarque-Bera Test	4.597268	3.503094	7.841147
Probability	0.100396	0.173505	0.01983 **

*, **, *** indicate significance at 10%, 5%, and 1%, respectively. HEC is health insurance medical expense expenditures. GDP is Gross Domestic Product. DEP is the dependent population of the insured.
